# Biological Synthesis of Low Cytotoxicity Silver Nanoparticles (AgNPs) by the Fungus *Chaetomium thermophilum*—Sustainable Nanotechnology

**DOI:** 10.3390/jof8060605

**Published:** 2022-06-04

**Authors:** Mariana Fuinhas Alves, Ariane Caroline Campos Paschoal, Tabata D’Maiella Freitas Klimeck, Crisciele Kuligovski, Bruna Hilzendeger Marcon, Alessandra Melo de Aguiar, Patrick G. Murray

**Affiliations:** 1 Shannon Applied Biotechnology Centre, Department of Applied Science, Faculty of Applied Sciences and Technology, Moylish Campus, Technological University of the Shannon: Midlands Midwest, Moylish, V94 EC5T Limerick, Ireland; 2 Laboratório de Biologia Básica de Células-Tronco, Instituto Carlos Chagas, FIOCRUZ Paraná, Curitiba 81350-010, PR, Brazil; arianepaschoal@hotmail.com (A.C.C.P.); crisciele.kuligovski@fiocruz.br (C.K.); bruna.marcon@fiocruz.br (B.H.M.); 3 Rede de Plataformas Tecnológicas FIOCRUZ-Plataforma de Microscopia, Instituto Carlos Chagas, FIOCRUZ Paraná, Curitiba 81350-010, PR, Brazil; tabata.klimeck@gmail.com; 4 Rede de Plataformas Tecnológicas FIOCRUZ-Bioensaios em Métodos Alternativos em Citotoxicidade, Instituto Carlos Chagas, FIOCRUZ Paraná, Curitiba 81350-010, PR, Brazil

**Keywords:** biosynthesis, *Chaetomium thermophilum*, cytotoxicity, fungus, silver nanoparticles

## Abstract

Fungal biotechnology research has rapidly increased as a result of the growing awareness of sustainable development and the pressing need to explore eco-friendly options. In the nanotechnology field, silver nanoparticles (AgNPs) are currently being studied for application in cancer therapy, tumour detection, drug delivery, and elsewhere. Therefore, synthesising nanoparticles (NPs) with low toxicity has become essential in the biomedical area. The fungus *Chaetomium thermophilum* (*C. thermophilum*) was here investigated—to the best of our knowledge, for the first time—for application in the production of AgNPs. Transmission electronic microscopy (TEM) images demonstrated a spherical AgNP shape, with an average size of 8.93 nm. Energy-dispersive X-ray spectrometry (EDX) confirmed the presence of elemental silver. A neutral red uptake (NRU) test evaluated the cytotoxicity of the AgNPs at different inhibitory concentrations (ICs). A half-maximal concentration (IC_50_ = 119.69 µg/mL) was used to predict a half-maximal lethal dose (LD_50_ = 624.31 mg/kg), indicating a Global Harmonized System of Classification and Labelling of Chemicals (GHS) acute toxicity estimate (ATE) classification category of 4. The fungus extract showed a non-toxic profile at the IC tested. Additionally, the interaction between the AgNPs and the Balb/c 3T3 NIH cells at an ultrastructural level resulted in preserved cells structures at non-toxic concentrations (IC_20_ = 91.77 µg/mL), demonstrating their potential as sustainable substitutes for physical and chemically made AgNPs. Nonetheless, at the IC_50_, the cytoplasm of the cells was damaged and mitochondrial morphological alteration was evident. This fact highlights the fact that dose-dependent phenomena are involved, as well as emphasising the importance of investigating NPs’ effects on mitochondria, as disruption to this organelle can impact health.

## 1. Introduction

Over-exploitation of natural resources and exponential human growth are the roots of modern social concerns [1]. In this context, sustainable consumption and production patterns, extensively described by the United Nations in the Sustainable Development Agenda as Goal 12, have inevitably become needed [2]; hence, the growing attention given to sustainable development and the pressing need to explore sustainable options [3]. As a result, physical and chemical processes are gradually being replaced by biological ones. This is not different in the nanotechnology field, as the biological route to synthesising nanoparticles (NPs) offers the benefits of environmental compatibility, scalability, and low or reduced production costs [4,5]. Also, the use of biological organisms as NP biofactories minimizes the use of hazardous chemicals, generating fewer or non-toxic end-products and, consequently fewer unwanted byproducts [6].

Silver nanoparticles (AgNPs) have been used in different fields of biotechnology; for example, to enhance the effectiveness of antibiotics and increase antibacterial activity by killing pathogenic and multiple-drug-resistant bacteria [7]. Additionally, they have been used to inhibit the viability of cancer cell lines [8,9]. Moreover, they have wide applications due to their antibacterial, antifungal, antiviral, anti-inflammatory, anti-angiogenic, and anticancer properties [10,11,12]. The advantages of using fungi rather than other microorganisms in NP synthesis include their metal tolerance and bioaccumulation capacity, their economic viability, and their suitability for handling biomass during downstream of processing and large-scale production [4].

Different fungi species, such as *Aspergillus fumigatus* (*A. fumigatus*), *Cladosporium halotolerans*, *Fusarium oxysporum* (*F. oxysporum*), *Penicillium italicum*, and *Trichoderma longibrachiatum* have been successfully used to synthesise AgNPs [8,10,13,14,15]. However, to the best of our knowledge, the species *Chaetomium thermophilum* (*C. thermophilum*) has not yet been investigated for NP synthesis. Hence, this study aimed to investigate the utilisation of their fungal metabolites in the extracellular synthesis of AgNPs. 

Regardless of the benefits of nanotechnology advances, nanomaterials’ physicochemical properties are still a source of concern with respect to the risks related to the production process, safety, and other environmental issues [16,17]. Furthermore, as human exposure to NPs is inevitable, it is crucial to understand their interactions with cellular systems and their toxicological impact [18]. Hence, toxicology research has been gaining significant attention. 

Several toxicity tests are available nowadays, such as cytotoxicity, neurotoxicity, genotoxicity, and ecotoxicity tests. Nevertheless, the Interagency Coordinating Committee on the Validation of Alternative Methods (ICCVAM) states that acute oral toxicity is usually the first tested in order to assess chemical hazards regarding classification, labelling, risk assessment, diagnosis, treatment, and prognosis toward chemical exposure [19]. 

Acute oral toxicity tests can be investigated in vivo or in vitro. However, for the implementation of alternative methodologies to use with animals, in vitro tests are essential, especially for stages of initial refinement of new substances with promising applications [19,20]. In this context, Balb/c 3T3 NIH (murine fibroblast) cells are recommended substrates for in vitro acute cytotoxicity testing [19,20]. This cell line has already been used to predict the cytotoxicity of AgNPs, and they have been proven to be more sensitive and accurate in toxicological evaluation than in vivo studies [21,22].

In vitro studies have reported that the interaction between AgNPs and cell cultures can cause diverse cytotoxicity outcomes, depending on the physical and chemical nature of the AgNPs and the cell lineage [12]. Toxicology studies have reported cytotoxicity effects, such as damage to the cell membrane and, consequently, alteration in the cell permeability, as well as severe morphological changes, especially in the mitochondria, leading to the impairment of this organelle [23,24,25]. Thus, there is a pressing need to predict nanomaterials’ toxicological impacts and to establish use of efficient, safe, reliable, and non-toxic NPs. The purpose of this study was, therefore, to: (1) investigate the ability of metabolites of the fungus *C. thermophilum* to synthesise AgNPs, (2) estimate AgNPs’ cytotoxicity using the neutral red uptake (NRU) assay, and (3) evaluate the interaction of AgNPs with the Balb/c 3T3 NIH cell line and further investigate potential safety hazards associated with biotechnological applications. The results of this study demonstrate the successful biological synthesis of AgNPs. The NRU cytotoxicity test predicted a lethal dose (LD_50_) value that indicated a Global Harmonized System of Classification and Labelling of Chemicals (GHS) category of 4 (300 mg/kg to 2000 mg/kg). Furthermore, preserved cell structures were observed following the interaction of AgNPs and Balb/c 3T3 NIH cells at an inhibitory concentration (IC) of IC_20_ and with an evident mitochondrial morphological alteration at IC_50_. These results highlight the applicability of the fungi system as a source of bio-nanomaterials with low cytotoxicity, low cost, and less impact on the environment that may eventually lead to sustainable development in the green nanotechnology field.

## 2. Materials and Methods

### 2.1. Production of Chaetomium thermophilum Cell-Free Extract 

The production of cell-free extract of the fungus *Chaetomium thermophilum var. thermophilum* (*C. thermophilum*), Centraal Bureau voor Schimmelcultures (CBS) collection number 143.50, was adapted from AbdelRahim et al., Hamedi et al., and Katapodis et al. [26,27,28]. The fungus was cultivated, for three days, at 45 °C in sterile *C. thermophilum* minimal agar (CTMA), composed of 0.10 g/L calcium chloride dihydrate (CaCl_2_·2H_2_O, Honeywell, Seelze, Germany), 15.00 g/L magnesium sulphate heptahydrate (MgSO_4_·7H_2_O, VWR, Dublin, Ireland), following chemicals were bought from Sigma-Aldrich (St. Louis, MO, USA): 30 mL/L corn steep liquor, 1.00 g/L yeast extract, 3.00 g/L potassium phosphate monobasic (KH_2_PO_4_), 2.00 g/L potassium phosphate dibasic (K_2_HPO_4_), 0.70 g/L noble agar. Trace mineral salts chemicals were bought from Sigma-Aldrich (St. Louis, MO, USA): 5.00 mg/L iron (II) sulphate heptahydrate (FeSO_4_·7H_2_O), 1.40 mg/L zinc sulfate heptahydrate (ZnSO_4_·7H_2_O), 1.60 mg/L manganese (II) sulfate tetrahydrate (MnSO_4_·4H_2_O), 0.20 mg/L cobalt (III) chloride hexahydrate (CoCl_3_·6H_2_O). In aseptic conditions, three samples (taken from the mycelia mat borders to guarantee actively growing fungal cells) were inoculated in 250 mL Erlenmeyer flasks with sterile *C. thermophilum* minimal medium (CTMM, without agar) at 45 °C and 120 rpm for five days. Fungal cells were filtered, washed thoroughly with sterile deionised water, dried, and weighed accurately. In order to induce secretion of secondary metabolites, the cells were transferred to sterile deionised water (stress liquid medium) with a ratio of 1 g of cells to 10 mL and incubated in the shaker for three days. The secreted fungal extract was separated from the fungus cells by muslin filtration, followed by centrifugation at 5000 rpm for 20 min at 25 °C and subsequent 0.22 µm membrane filtration. The *C. thermophilum* cell-free extract was stored at 4 °C until further use. 

### 2.2. Biological Synthesis and Characterisation of AgNPs 

#### 2.2.1. AgNP Synthesis

The AgNP synthesis activity level of the *C. thermophilum* cell-free extract was tested following the study by Alves and Murray [3]. Briefly, the cell-free extract was heated for 15 min at 90 °C; then, 127.40 mg/L silver nitrate (AgNO_3_, Sigma-Aldrich, St. Louis, MO, USA) was added for a total volume of 500 μL, and heating was continued for one hour at 90 °C. In the same reaction conditions, a reaction synthesis control was produced using ultrapure water and AgNO_3_. 

#### 2.2.2. AgNP Characterization

The resulting AgNPs were characterised based following the study by Alves and Murray [3]. Ultraviolet–visible spectrophotometry (UV-Vis, BioTek Synergy 4 Microplate Reader, Bad Friedrichshall, Germany) was first used to measure the localised surface plasmon resonance (LSPR) absorbance. AgNPs were scanned between 300 nm and 1000 nm, with 2 nm steps. Afterwards, transmission electron microscopy (TEM, JEOL JEM 2100 Field Emission Electron Microscope, Tokyo, Japan) was used to evaluate the shape and size distribution of the AgNPs. Samples were drop-coated onto Formvar carbon-coated copper grids with a 200 µm mesh size and dried over 24 h. Images were obtained using a JEOL JEM 2100 Field Emission Electron Microscope operated at 200 kV with a field emission electron gun equipped with a Gatan Ultrascan digital camera. An average of 200 NPs was recorded from several TEM images, with AxioVision Rel 4.8 software used to evaluate size distribution. Furthermore, the elemental composition was analysed using energy dispersive X-ray spectrometry (EDX, Hitachi 3000, Tokyo, Japan). Five microliters of the samples was drop-coated onto polished aluminium slides and dried in the oven at 60 °C for 1 h, thrice. A Hitachi 3000 electron microscope with EDX capability at 15 kV accelerating voltage and a working distance of 2 mm was used to obtain the ED spectra of the samples. 

### 2.3. Mammalian Cell Culture

Skin fibroblasts from murine embryo Balb/c mice 3T3 NIH (clone A31) cells (Balb/c 3T3 NIH cells, Rio de Janeiro Cell Bank, Rio de Janeiro, Brazil), the cells recommended by ICCVAM [29], were cultivated in routine medium containing high-glucose (4.5 g/L) Dulbecco’s Modification of Eagle’s Medium (DMEM, Gibco Invitrogen, Carlsbad, CA, USA) supplemented with non-heat-inactivated 10% fetal bovine serum (FBS, Gibco Invitrogen, Carlsbad, CA, USA) and 4 mM L-Glutamine (Gibco Invitrogen, Carlsbad, CA, USA) at 37 °C, 90% humidity, and 5.0% CO_2_/air [29,30].

### 2.4. Cytotoxicity Evaluation Profile in Mammalian Cell 

The cytotoxicity evaluation of the *C. thermophilum* AgNPs and of the *C. thermophilum* cell-free extract was based on the Organisation for Economic Co-operation and Development (OECD) guidelines from the Environment, Health and Safety Publications Series on Testing and Assessment No. 129 guidance document on using cytotoxicity tests to estimate starting doses for acute oral systemic toxicity tests [20]. In brief, the cells were plated into the inner wells of 96-well tissue culture microtiter plates at a density of 2.5 × 10^3^ cells (100 µL/well); the outer wells were filled with culture medium and then cultivated for 24 h at 37 °C, 90% humidity, and 5.0% CO_2_/air. After 24 h of incubation, the culture medium was removed. The AgNPs were diluted immediately prior to use with a solution of 4.5 g/L DMEM, 4 mM L-Glutamine (Gibco Invitrogen, Carlsbad, CA, USA), 100 IU/mL penicillin (Sigma-Aldrich, St. Louis, MO, USA), and 100 µg/mL streptomycin (Sigma-Aldrich, St. Louis, MO, USA), according to ICCVAM recommendations [31]. Eight serial dilutions of AgNPs ranging from 31.03 μg/mL to 460.32 μg/mL were prepared. For the AgNP serial dilution, the log-factor of 1.47 was used. Eight serial dilutions of the *C. thermophilum* cell-free extract ranging from 0.38 μg/mL to 1200 μg/mL were also prepared, with a serial dilution log factor of 3.16. Plates were incubated for 48 h at 37 °C, 90% humidity, and 5.0% CO_2_/air. After this period, Balb/c 3T3 NIH cells were stained with a neutral red (NR) medium composed of 25 µg/mL NR dye (Sigma-Aldrich, St. Louis, MO, USA). NR was extracted from cells using 250 µL/well of NR desorb solution (freshly prepared with 49 parts water, 50 parts ethanol, and 1 part glacial acetic acid, all bought from Sigma-Aldrich, St. Louis, MO, USA) over a period of 20 min in a shaker protected from light [32,33]. The optical density of the samples was measured at a wavelength of 540 nm using a Multi-Modo Synergy H1 (Biotek, Winooski, Vermont, EUA) spectrophotometer reader.

### 2.5. Evaluation of Balb/c 3T3 NIH Cells and AgNP Interaction by TEM

Balb/c 3T3 NIH were plated into 6-well tissue culture microtiter plates at a density of 7.5 × 104 cells/well in 3 mL of the routine medium and cultivated for 24 h at 37 °C, 90% humidity, and 5.0% CO_2_/air. After this period, the routine medium was discharged and 1.5 mL of a fresh one was added. The cells were submitted to three treatments: a control (cells without AgNPs), a non-toxic concentration (IC_20_), and the IC_50_. Plates were incubated for 6 h and rinsed with pre-warmed phosphate buffered saline (PBS, Sigma-Aldrich, St. Louis, MO, USA). The cells were kept in a fixing solution composed of 2.5% glutaraldehyde (Sigma-Aldrich, St. Louis, MO, USA) and diluted in 0.1 M sodium cacodylate buffer solution added over a period of 24 h. The cells were washed with 0.1 M sodium cacodylate buffer (Electron Microscopy Sciences) and post-fixed with 1% osmium tetroxide (Electron Microscopy Sciences, Hatfield, PA, USA), 0.8% potassium ferricyanide (Electron Microscopy Sciences, Hatfield, PA, USA), 5 mM calcium chloride, and 0.1 M sodium cacodylate buffer. After washing, the samples were dehydrated using a graded acetone series (30%, 50%, 70%, 90%, and 100%) and embedded using EMBed 812 resin (Electron Microscopy Sciences, Hatfield, PA, USA). Ultrathin sections of each sample were obtained using a Leica EM UC6 ultramicrotome (Leica, Wetzlar, Germany). The samples were contrast-stained with 5% uranyl acetate (Sigma-Aldrich, St. Louis, MO, USA) for 30 min and with lead citrate (Sigma-Aldrich, St. Louis, MO, USA) for 5 min, then analysed using a JEOL JEM1400-Plus TEM (JEOL, Tokyo, Japan) [30,33].

### 2.6. Statistical Analysis

All assays were run in triplicate. Data were expressed as means ± standard deviation (St. Dev). The software packages used to analyse the data generated in the characterisation process were Gen5, Microsoft Office Excel, and Quantax 70 Microanalysis. Cell viability based on optical density data was analysed in Microsoft Office Excel. Outliers were analysed with the statistics Grubbs test (available online at www.graphpad.com/quickcalcs/Grubbs1.cfm, accessed on 12 June 2021). GraphPad Prism^®^ 5.0 was used to create a sigmoidal dose–response (variable slope) with four parameters, rearranged in the Hill function. IC_20_, IC_50_, and IC_80_ were expressed graphically with mean and standard deviation. IC_50_ was used to predict LD_50_ using the formula: log LD_50_ (mg/kg) = 0.372 log IC_50_ (μg/mL) + 2.024 (R^2^ = 0.325) [30,31,32,33]. Once the LD_50_ was predicted, it was possible to classify the AgNPs according to the Globally Harmonized System of Classification and Labelling of Chemicals (GHS) classification [34]. The acceptance criteria of the assay followed ICCVAM guidelines [31]. Sodium dodecyl sulphate (SDS, Sigma-Aldrich, St. Louis, MO, USA) was used as the control drug, and the acceptance criteria of the assay also followed the ICCVAM guidelines [31,35].

## 3. Results

### 3.1. Production of Chaetomium thermophilum Cell-Free Extract

**Figure 1:**
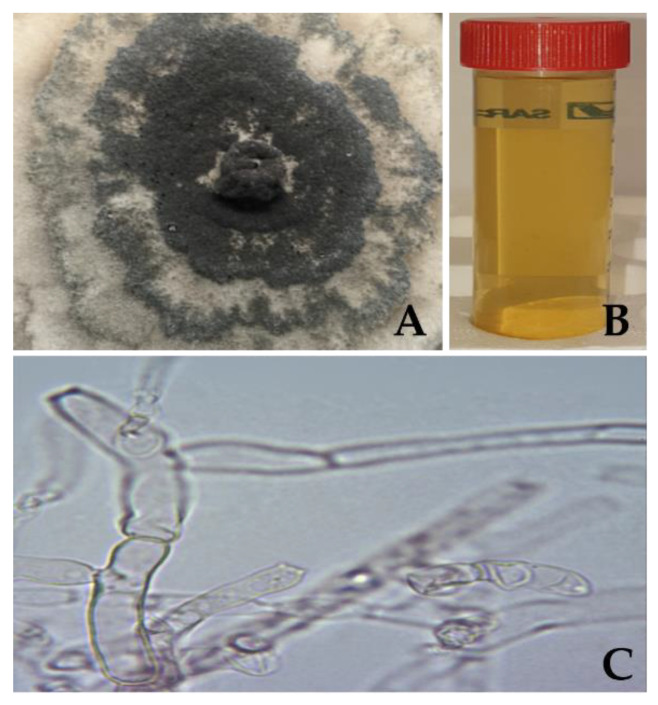
*C. thermophilum* biological characterisation. (**A**) Fungal growth in CTMA medium. (**B**) Fungal cell-free extract ready to be used in nanoparticles synthesis. (**C**) Microscope image of mycelia cells with 100× magnification.

The fungus *C. thermophilum* grows with septate hyphae that develop initially with a lightly hyaline colour (glass-like) and change over time to olivaceous (brownish olive) with thicker septate walls [36]. Photos of the fungus growth in CTMA plates and of the cell-free extract were taken, as shown in Figure 1A,B, and the mycelia cells were analysed using a microscope (VWR, Dublin, Ireland) with 100× magnification (Figure 1C).

### 3.2. AgNP Characterisation

**Figure 2:**
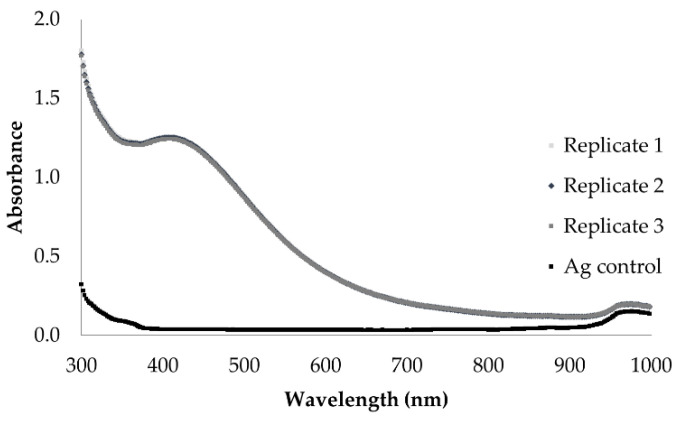
Biological AgNPs physicochemical characterisation. UV/Vis spectrophotometry analysis of the biologically synthesised AgNPs by the fungus *C. thermophilum*.

The LSPR absorbance in the wavelength region of 380 nm to 435 nm indicates AgNP synthesis. The UV-Vis spectrophotometry spectrum of the AgNPs displayed a maximum wavelength of 405 ± 1.15 nm, with a correspondent maximum absorbance value of 1.250 ± 0.01, demonstrating a different pattern from the spectrum of the synthesis reaction control (Figure 2). The AgNO_3_ control solution did not present an absorbance peak in the relevant UV-Vis region.

**Figure 3:**
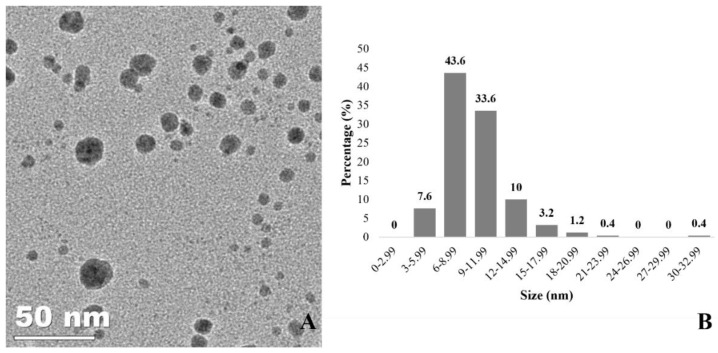
Biological AgNPs physicochemical characterisation. (**A**) TEM image (60000× magnification, 50 nm scale) showing spherical shaped AgNPs synthesised by the fungus *C. thermophilum*. (**B**) Size distribution, with the measurement obtained using ImageJ software, with 8.93 ± 2.29 nm average size.

Figure 3 shows a 60,000× magnification TEM image of the spherical-shaped AgNPs synthesised. Statistical analysis revealed that the AgNPs’ size distribution ranged from 4.72 nm to 30.73 nm, with an average size of 8.93 ± 2.29 nm. Furthermore, EDX analysis (data not shown) was carried out on the samples, and the presence of elemental silver was confirmed at 3 keV where AgNPs were identified in the TEM.

### 3.3. NRU Cytotoxicity Evaluation

**Figure 4:**
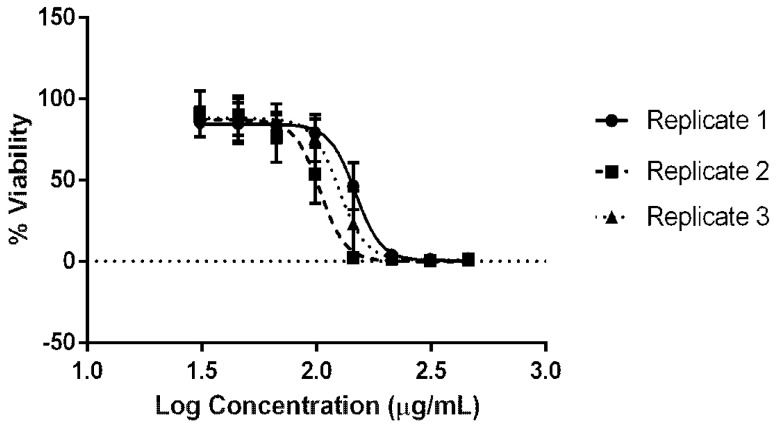
Cytotoxicity evaluation of the AgNPs biologically synthesised by the fungus *C. thermophilum*: the AgNP dose–response curves (Hill function fit) of the NRU assay using the skin fibroblasts from the murine embryo Balb/c 3T3 NIH cell line.

**Figure 5:**
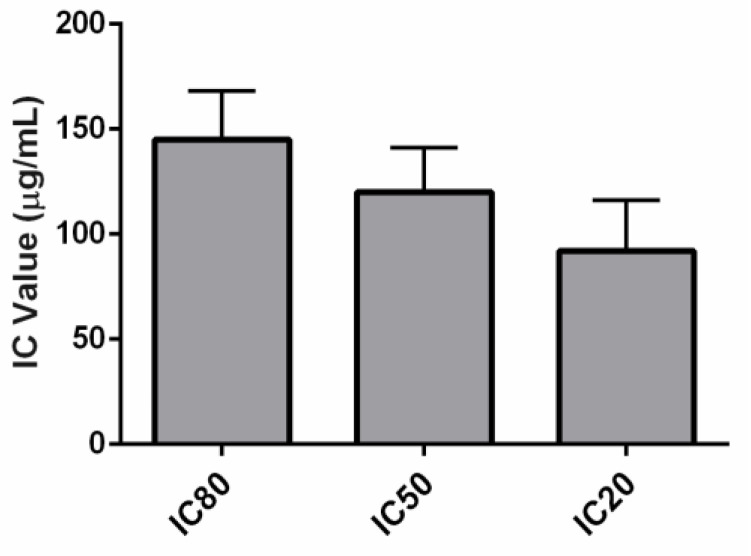
Cytotoxicity evaluation of the AgNPs biologically synthesised by the fungus *C. thermophilum* using the NRU assay with the skin fibroblasts from the murine embryo Balb/c 3T3 NIH cell line: IC values.

The cytotoxicity of the AgNPs synthesised using *C. thermophilum* cell-free extract was evaluated using the NRU following OECD guidelines. AgNP concentrations ranging from 31.03 µg/mL to 460.32 µg/mL (dilution log factor: 1.47) were tested. Dose–response curves were obtained using GraphPad Prism^®^ and Excel software (Figure 4). The average IC values were as follows: IC_20_ = 91.77 ± 24.24 µg/mL, IC_50_ = 119.69 ± 21.15 µg/mL, and IC_80_ = 144.92 ± 23.22 µg/mL. The IC_50_ was used to predict an LD_50_ value of 624.31 ± 41.87 mg/kg, suggesting that the AgNPs synthesised might belong to GHS category 4 with regard to inducing acute toxicity (Figure 5 and Table 1).

**Figure 6:**
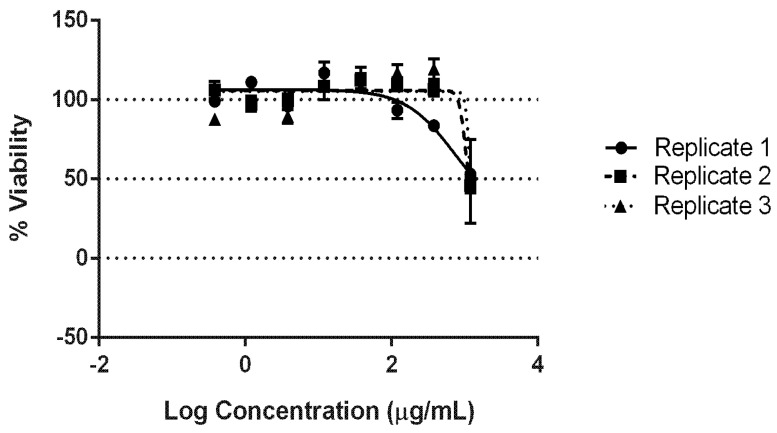
Cytotoxicity evaluation of the *C. thermophilum* cell-free-extract fungal metabolites: dose–response curves (Hill function fit) of the NRU assay using skin fibroblasts from the murine embryo Balb/c 3T3 NIH cell line.

Additionally, the cytotoxicity of the *C. thermophilum* cell-free extract was analysed to evaluate potential toxicity derived from fungal metabolites. Concentrations ranging from 0.38 µg/mL to 1200 µg/mL (dilution log factor: 3.16) were tested. At 1200 µg/mL, the highest IC tested for the extract (2.5 times higher than the maximum AgNP concentration tested), a maximum of 50% of the cells were killed (Figure 6.). Thus, the IC_50_ and, consequently, the LD_50_ were not statistically calculated, nor was the GHS acute toxicity classification, as this was based on the LD_50_ values. Hence, the extract cytotoxicity was beyond the evaluated cytotoxicity range of this test. Therefore, the *C. thermophilum* cell-free extract was effectively non-toxic at the IC tested.

### 3.4. AgNPs-Balb/c 3T3 NIH Cells Interaction

**Figure 7:**
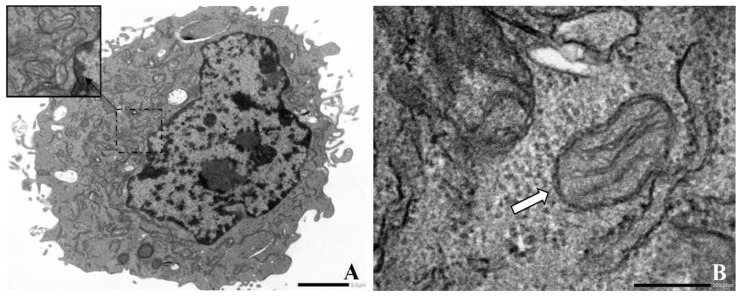

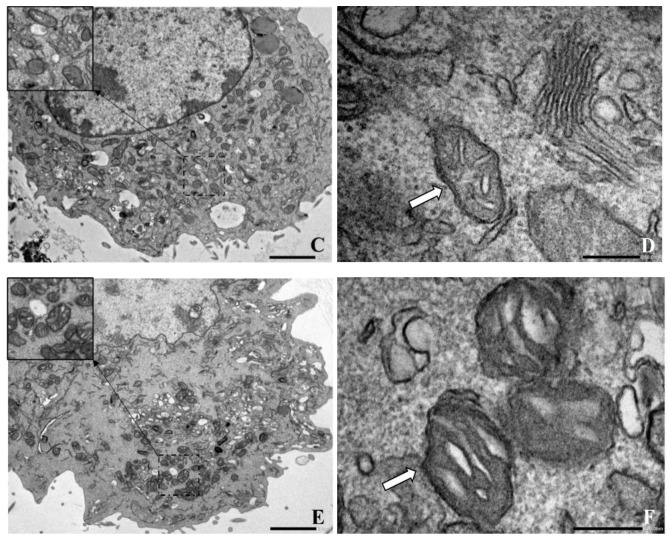
TEM ultrastructural evaluation of the interaction between the AgNPs biologically synthesised by the fungus *C. thermophilum* cell-free extract and skin fibroblasts from the murine embryo Balb/c 3T3 NIH cell line. (**A**) Negative control (Balb/c 3T3 NIH cells without treatment). (**B**) Mitochondria organelle of Balb/c 3T3 NIH cells without treatment. (**C**) Balb/c 3T3 NIH cells exposed to a non-toxic AgNP concentration (IC_20_ = 91.77 ± 24.24 µg/mL), demonstrating no significant cell damage or alteration at the ultrastructural level. (**D**) Mitochondria organelle of Balb/c 3T3 NIH cells exposed to a non-toxic AgNP concentration (IC_20_). (**E**) Balb/c 3T3 NIH cells exposed to an IC_50_ (119.69 ± 21.15 µg/mL), showing that the cell cytoplasm was damaged. (**F**) Mitochondria organelle of Balb/c 3T3 NIH cells exposed to an IC_50_ with an evident morphological alteration.

The interaction between the AgNPs and the Balb/c 3T3 NIH cells was evaluated at an ultrastructural level in three different scenarios: (1) a control in which the cells were not exposed to the AgNPs, (2) a non-cytotoxic AgNP concentration (IC_20_), and (3) the IC_50_. Compared to the control (Figure 7A,B), most cells had a preserved ultrastructure at the IC_20_ (Figure 7C,D). Hence, no evidence of cell alteration at the ultrastructural level was detected. However, at the IC_50_ (Figure 7E,F), the cell cytoplasm was damaged, and mitochondrial morphological alteration was evident.

## 4. Discussion

Research involving silver nanostructures has expanded rapidly due to their promising applications within the biomedicine and biotechnology fields. However, despite their exceptional physicochemical properties, concerns related to environmental toxicity and health-related hazards exist [16,17]. Therefore, efforts have been made in order to develop eco-friendly approaches to synthesising AgNPs. Overall, the advantage of the biological route involves the process simplicity, the use of non-hazardous reducing and stabilising reagents, and comparatively low production costs [4,5]. 

This study showed that the fungus *C. thermophilum* cell-free extract can biologically synthesise spherically shaped AgNPs with an average size of 8.93 ± 2.29 nm. The genus *Chaetomium* has been previously investigated with regard to NP synthesis. The *Chaetomium* species *C. globosum* and *C. cupreum* were used to produce copolymer NPs with bioactive compounds from their crude methanol extract [37]. Furthermore, the species *C. globosum* was used in iron NP production [38]. To the extent of our knowledge, the species *C. thermophilum* has not been yet investigated for NP synthesis. 

*C. thermophilum* is a moderately thermophilic fungus with slow growth at 35 °C but with optimum growth temperatures in the 40 to 55 °C range [36]. Its genome sequence was first described by Amlacher et al. [39]. It has since been used as a model organism system for biophysical research. Recently, this fungus was involved in high-temperature protein adaptation studies that demonstrated that protein glycosylation and deglycosylation are the mechanisms that allow their thermophily [40]. Furthermore, different authors have investigated this species’ enzymes in the breakdown of lignocellulose for biofuel-renewable biomass, as well as for their potential to degrade cellulosic waste [18,41].

In vitro cytotoxicity tests are used to determine the toxicity of substances in cell lines as an alternative to direct animal testing. In vitro systems are relevant models when investigating the common toxicity mechanisms of AgNPs because they are cost-effective and allow direct assessment of NPs, providing valuable data for the screening of toxicity [12]. The advantages relate to the costs, the time required to obtain the final results, and ethical issues [42]. The NRU assay is an example of an in vitro cytotoxicity test. The OECD published a guideline document (No. 129 from the Environment, Health and Safety Publications Series on Testing and Assessment) for this test in 2010. The cell lines Balb/c 3T3 (murine fibroblast) or NHK (normal human keratinocytes) are used to measure toxicity as a concentration-dependent reduction of the chemical NR cell uptake after the substance’s test exposure [20]. Obtaining cytotoxicity results with a sensitive cell line, such as Balb/c 3T3, is a valuable screening approach. However, additional tests must be taken, not only to assess toxicity mechanisms but also to assess the effectiveness in other models of biomedical relevance.

It is important to highlight that the cytotoxicity effects depend on the AgNPs’ physical and chemical nature, concentration, and incubation time; the presence of serum and, hence, protein coronas; the presence of ion release, agglomeration in the cell medium, and intracellular localization; the cell lineage; and other factors [12]. In this context, colourimetry tests, such as tests involving NRU, 3-(4,5-dimethylthiazol-2-yl)-2,5-diphenyl-2H-tetrazolium bromide (MTT), and (3-(4,5-dimethylthiazol-2-yl)-5-(3-carboxymethoxyphenyl)-2-(4-sulfophenyl)-2H-tetrazolium) (MTS), are often used in the cytotoxicity evaluation of AgNPs. 

The GHS establishes that the acute toxic estimate (ATE) via the oral exposure route can be used to classify substances into five categories based on the LD_50_ values. A lower LD_50_ (mg/kg) is an indicator of greater toxicity [34]. This study successfully employed the OECD-based methodology (Figure 4). The NRU cytotoxicity assay was used to calculate the value of the IC_50_ (119.69 ± 21.15 µg/mL) of the biological AgNPs produced by the fungus *C. thermophilum* and to predict an LD_50_ value of 624.31 ± 41.87 mg/kg. Hence, this indicates a GHS ATE classification as category 4 (Figure 5). For contextual reference, the NRU assay control drug, sodium dodecyl sulphate (SDS), which is used in cleaning and hygiene products, has a rat oral LD_50_ of 977 mg/kg, also fitting into class 4 (300 mg/kg to 2000 mg/kg) [43]. 

The AgNPs biologically synthesised by the fungus *C. thermophilum* were significantly less toxic than chemically synthesised AgNPs, which have reported IC_50_ values ranging from 2.20 μg/mL to 10 μg/mL, indicating higher toxicity [22,23,44,45]. Importantly, they were also found to be 4 to 30 times less toxic than other AgNPs synthesised with different species (Table 2) [45,46,47]. It is essential to highlight that AgNPs are currently being explored for applications in cancer therapy, tumour detection, drug delivery, wound dressing, and elsewhere [46,48,49,50,51,52]. Therefore, synthesising NPs with lower toxicity, as demonstrated in this study, may lead to an expansion in the range of applications in the biomedical field.

Although multiple species of fungi have been claimed to synthesise AgNPs, some of these species are pathogenic. For example, *F. oxysporum* was reported to synthesise well-dispersed, spherically shaped AgNPs with sizes ranging from 5 nm to 13 nm. However, this species is responsible for soil-borne diseases that are extremely difficult to control, affecting food plants such as tomatoes, bananas, and onions [8,46]. Another example is the fungus *A. fumigatus*, described as responsible for the extracellular synthesis of extremely small (0.68 nm) cube-shaped AgNPs [15]. Nonetheless, this genus is also responsible for a parenchymal lung disease called aspergillosis [56]. 

While fungi produce a large variety of secondary metabolites, which may be critical for NP synthesis and stabilisation, they are also known to produce harmful self-preservation chemicals, including mycotoxins. In small concentrations, these low-molecular-weight compounds can be toxic to vertebrates and other animal groups [57]. Hence, fungal extracts also require toxicity evaluation for any proposed biological applications of NPs synthesised using fungi. 

In this study, it was demonstrated that the *C. thermophilum* cell-free extract used to synthesise the AgNPs was effectively non-toxic at the maximum IC tested (1200 µg/mL, Figure 6). Similar results were obtained in a cytotoxicity study of culinary–medicinal mushroom aqueous extract using the NRU assay with Balb/c 3T3 NIH cells. The non-toxic fungal extracts of *Ganoderma lucidum* (IC_50_ = 1350 µg/mL), *Ganoderma neo-japonicum* (IC_50_ = 1780 µg/mL), *Hericium erinaceus* (IC_50_ = 3530 µg/mL), *Lignosus rhinocerotis* (IC_50_ = 5600 µg/mL), and others were reported [58]. 

Furthermore, this study evaluated the interaction between the AgNPs and the Balb/c 3T3 NIH cells at an ultrastructural level (Figure 7). The different mechanisms by which AgNPs induce cell death include Ag ion release, disruption of cell membrane integrity, oxidative stress, protein or deoxyribonucleic acid (DNA) damage, generation of reactive oxygen species, and apoptotic cell death [59,60]. The ultrastructural damage caused by NP–cell interaction is often identified using TEM. 

The TEM study demonstrated that most cells had preserved ultrastructures at the IC_20_ (91.77 ± 24.24 µg/mL, Figure 7C,D). However, at the IC_50_ (119.69 ± 21.15 µg/mL), the cell cytoplasm was damaged (Figure 7E). The mitochondrial morphological alteration was evident in the swelling of the inner membrane (Figure 7F). Similar results were found after analysing the interaction between mitochondria and chemically synthesised AgNPs. For example, a significant decrease in the mitochondrial membrane potential, adenosine diphosphate (ADP)-induced depolarisation, and respiratory control ratio were reported in rat liver mitochondria exposed to 40 nm and 80 nm of AgNPs. The function impairment was mainly attributed to changes in the membrane permeability [25]. Furthermore, exposure of AgNPs and titanium NPs to rat liver mitochondria was also demonstrated to lower the respiratory control ratio and induce mitochondrial swelling [61]. Additionally, the interaction of adult Wistar rats with chemically produced AgNPs (10 ± 4 nm) at a low dosage (0.2 mg/kg b.w.) resulted in mitochondrial swelling and cristolysis (damage of cristae) caused by silver nano-granules in the brain [24].

Mitochondria are cytoplasmic, double- membrane-bound organelles known to play an essential role in cellular energy production and to participate in calcium signalling, cell growth, differentiation, and death [62]. Moreover, mitochondria dysfunction has been related to many diseases, including neurodegenerative disorders; Huntington’s, Parkinson’s, and Alzheimer’s diseases; epilepsy; schizophrenia; and Leigh syndrome [63,64,65]. Thus, it is essential to investigate the effects of NPs on mitochondria at a structural level, as disruption of this organelle can result in health effects.

## 5. Conclusions

As sustainable development awareness has grown, there has been a shift in focus to the use of biological sources. In the nanotechnology field, fungi have been investigated for the production of AgNPs in an environmentally friendly manner. This study reported the successful biological synthesis of AgNPs using the fungus *C. thermophilum* and is, to the best of our knowledge, the first time this fungus has been studied with regard to NPs production. Furthermore, it demonstrated low AgNP cytotoxicity towards a reference mammalian cell line, Balb/c 3T3 NIH. Additionally, the interaction of the AgNPs with this cell line showed preserved cell structures at non-toxic IC_20_. Therefore, the biologically synthesised AgNPs described in this study have potential as sustainable substitutes for physically and chemically made AgNPs; hence, they are a step in the right direction in achieving sustainable development in the nanotechnology field.

## Figures and Tables

**Table 1:** Cytotoxicity evaluation of the AgNPs biologically synthesised by the fungus *C. thermophilum* using the NRU assay with the skin fibroblasts from the murine embryo Balb/c 3T3 NIH cell line: IC values, the predicted LD_50_ (which, given all at once, could cause the death of 50% of a group of test animals) and the GHS for the AgNPs’ cytotoxic effects.

IC		Predicted LD_50_	GHS
µg/mL		mg/kg	
**IC_80_**	144.92 ± 23.33	624.31 ± 41.87	4
**IC_50_**	119.69 ± 21.15		
**IC_20_**	91.77 ± 24.24		

**Table 2:** Comparative IC_50_ of AgNPs synthesised in different ways.

AgNP SYNTHESIS	IC_50_	CYTOTOXIC TEST	CELL LINEAGE	REF.
	µg/mL			
Biological: *Fusarium semitectum*	260.00	MTT	HGF human fibroblast	[45]
Biological: *Gloeophyllum striatum*	28.76	MTT	L929 mouse fibroblasts	[47]
Biological: *Streptomyces* sp.	64.50	MTT	L929 mouse fibroblasts	[53]
Biological: *Streptomyces xinghaiensis*	4.0	MTT	BALB/c 3T3 fibroblasts	[54]
Biological: *Canna edulis*	18.00	NRU/MTT	L929 mouse fibroblasts	[6]
Chemical: PVP-AgNP	2.80	NRU	BALB/c 3T3 fibroblasts	[22]
Chemical: PVP-AgNP	2.80	NRU	BALB/c 3T3 fibroblasts	[21]
Chemical: Na^3^C^6^H^5^O^7^-AgNP	10.00 *	MTS	BALB/c 3T3 fibroblasts	[55]
Chemical: Na^3^C^6^H^5^O^7^-AgNP	7.00	NRU	NCTC 929 fibroblast	[44]
Biological: *Chaetomium thermophilum*	119.69	NRU	Balb/c 3T3 fibroblast	Present study

* Substantial numbers of dead cells (56.8%).
